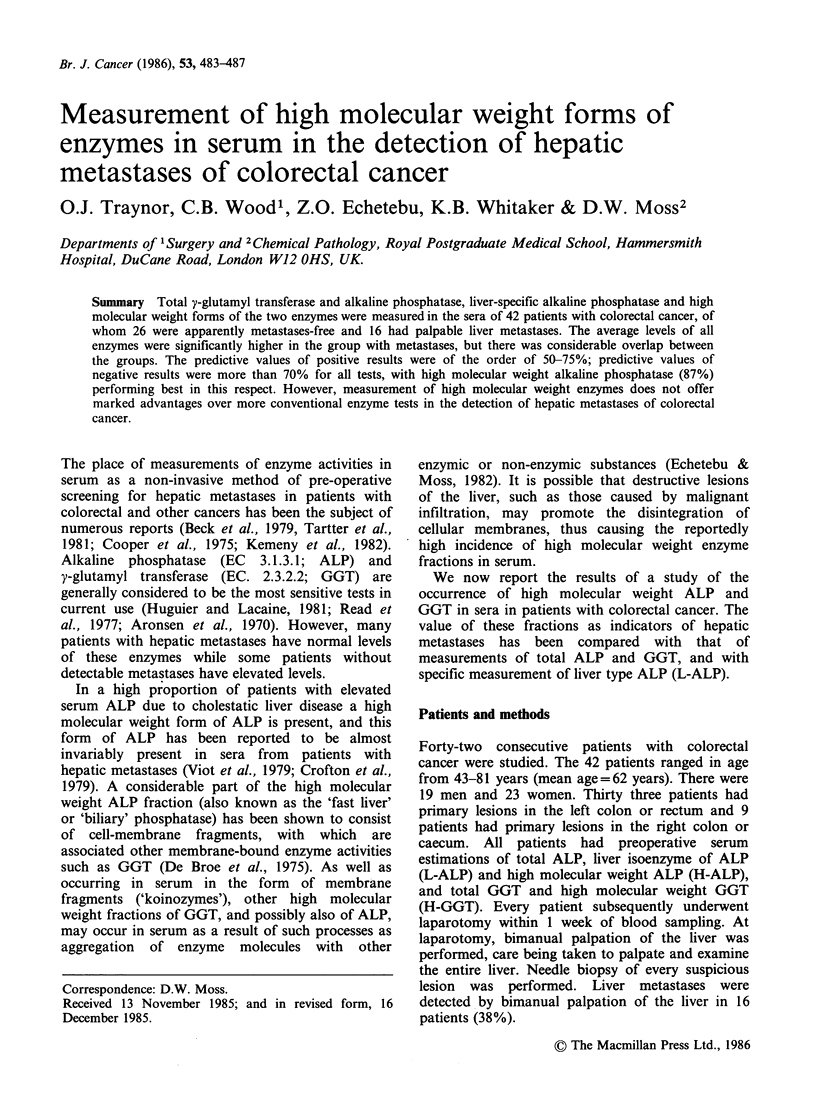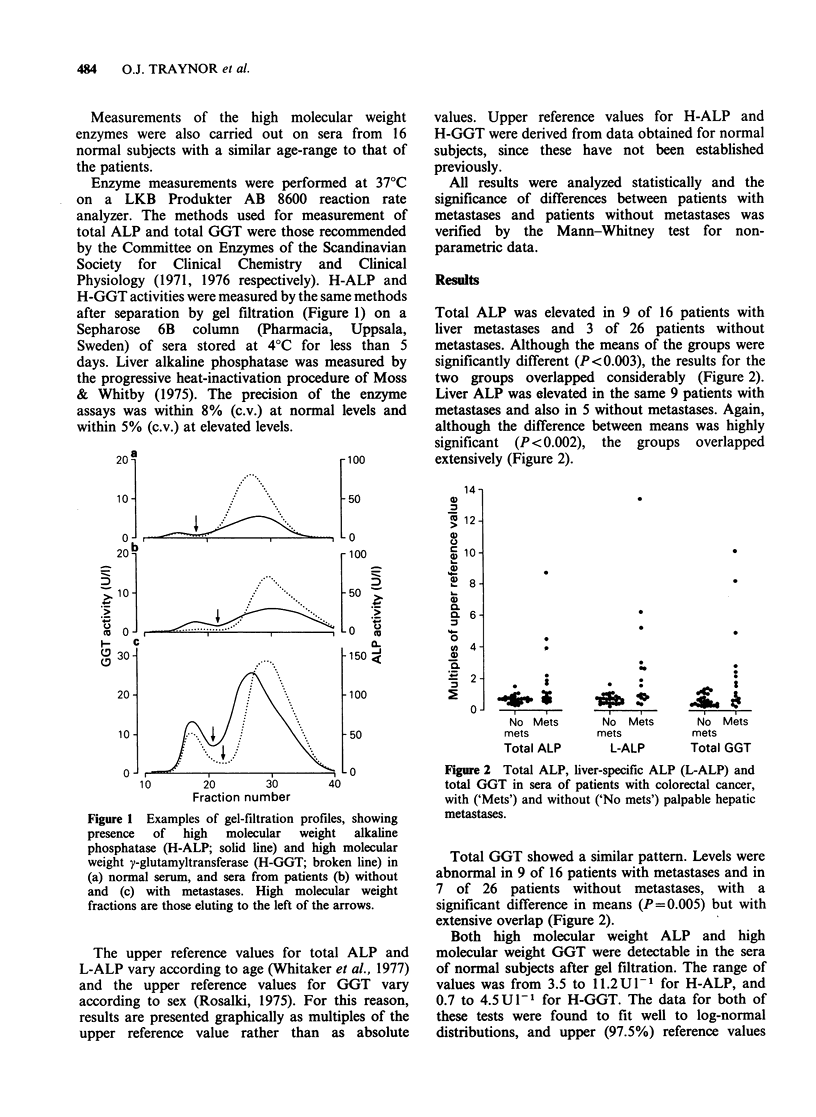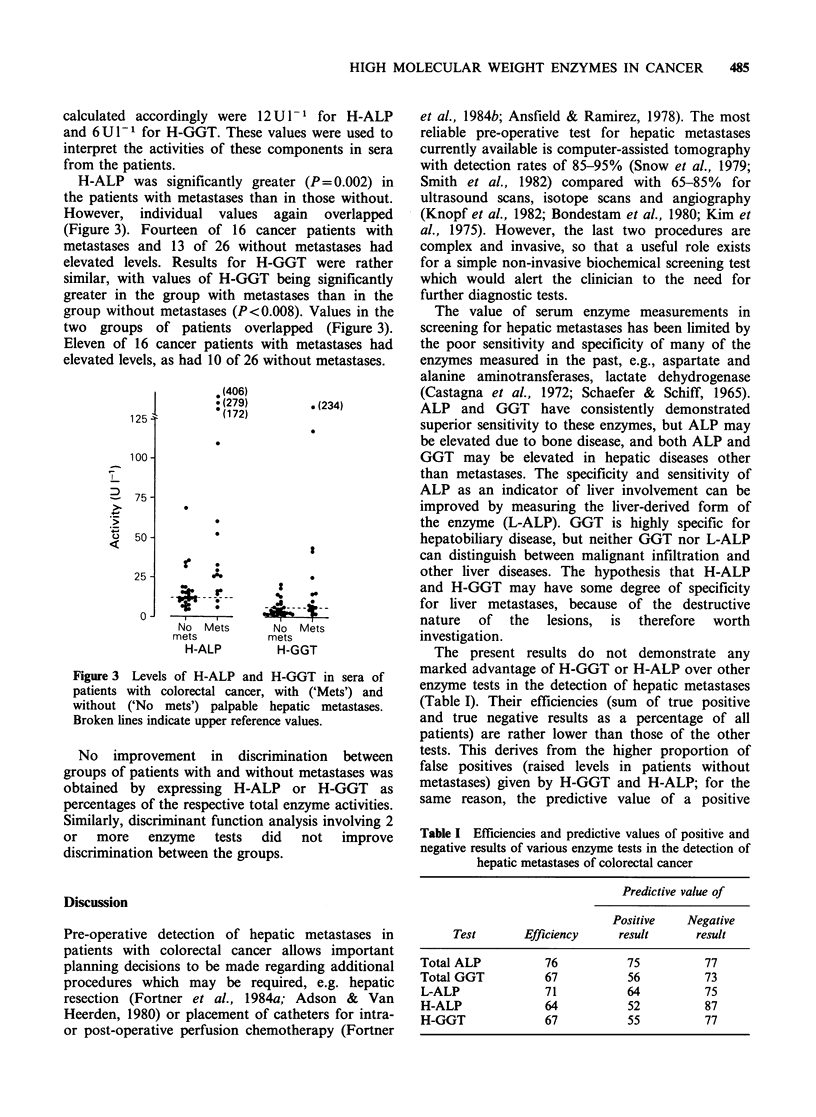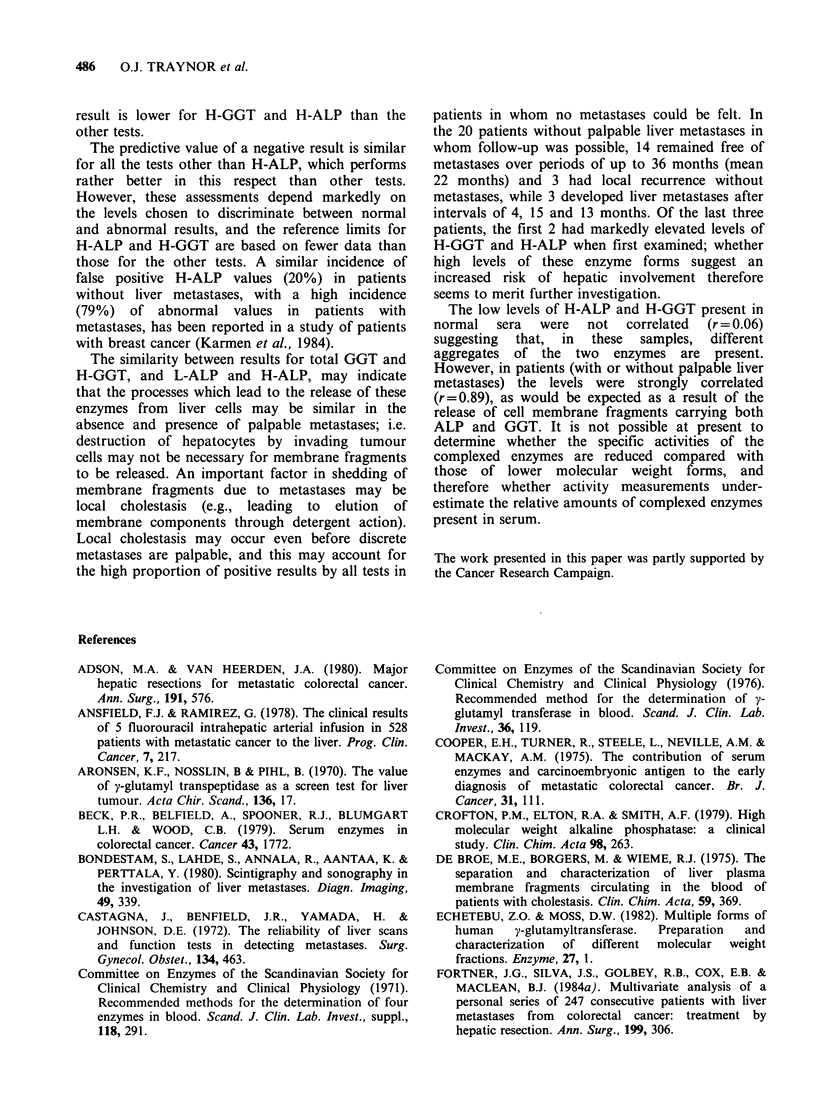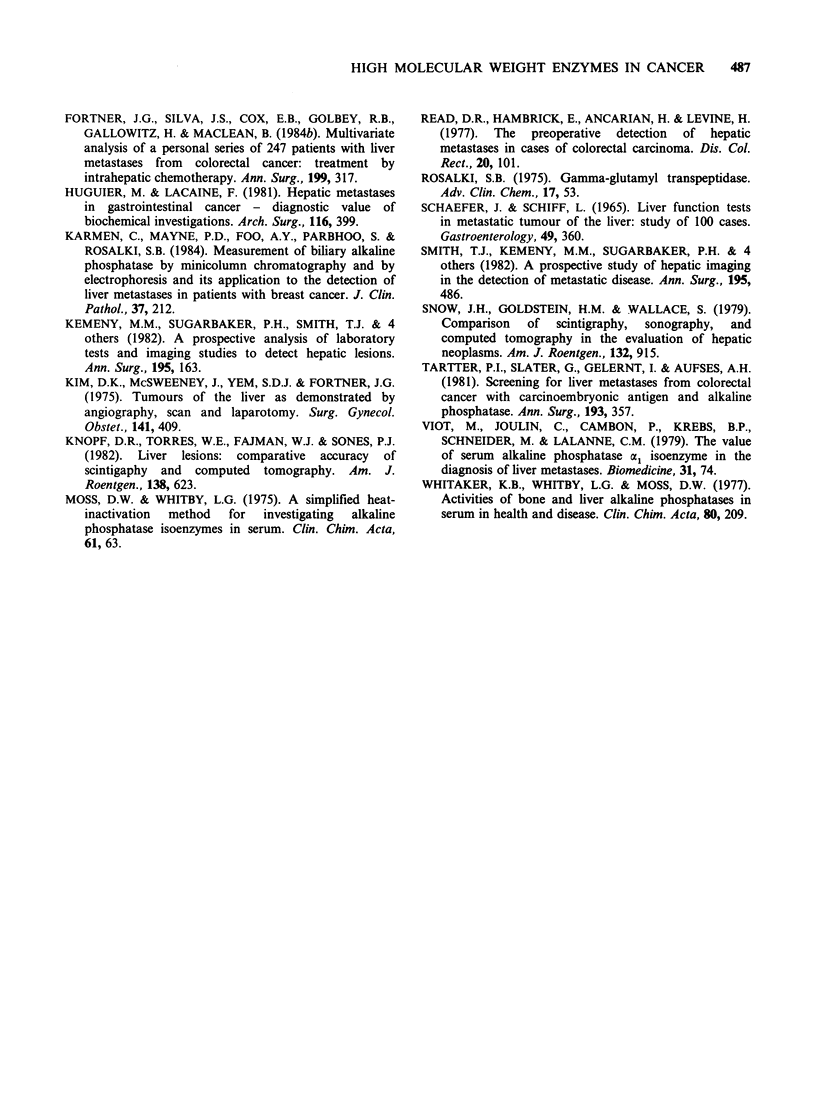# Measurement of high molecular weight forms of enzymes in serum in the detection of hepatic metastases of colorectal cancer.

**DOI:** 10.1038/bjc.1986.76

**Published:** 1986-04

**Authors:** O. J. Traynor, C. B. Wood, Z. O. Echetebu, K. B. Whitaker, D. W. Moss

## Abstract

Total gamma-glutamyl transferase and alkaline phosphatase, liver-specific alkaline phosphatase and high molecular weight forms of the two enzymes were measured in the sera of 42 patients with colorectal cancer, of whom 26 were apparently metastases-free and 16 had palpable liver metastases. The average levels of all enzymes were significantly higher in the group with metastases, but there was considerable overlap between the groups. The predictive values of positive results were of the order of 50-75%; predictive values of negative results were more than 70% for all tests, with high molecular weight alkaline phosphatase (87%) performing best in this respect. However, measurement of high molecular weight enzymes does not offer marked advantages over more conventional enzyme tests in the detection of hepatic metastases of colorectal cancer.


Br. J. Cancer (1986), 53, 483–487

### Measurement of high molecular weight forms of enzymes in serum in the detection of hepatic metastases of colorectal cancer

O.J. Traynor, C.B. Wood[1], Z.O. Echetebu, K.B. Whitaker & D.W. Moss[2]

*Departments of [1]Surgery and [2]Chemical Pathology, Royal Postgraduate Medical School, Hammersmith Hospital, DuCane Road, London W12 0HS, UK.*

**Summary** Total γ-glutamyl transferase and alkaline phosphatase, liver-specific alkaline phosphatase and high molecular weight forms of the two enzymes were measured in the sera of 42 patients with colorectal cancer, of whom 26 were apparently metastases-free and 16 had palpable liver metastases. The average levels of all enzymes were significantly higher in the group with metastases, but there was considerable overlap between the groups. The predictive values of positive results were of the order of 50–75%; predictive values of negative results were more than 70% for all tests, with high molecular weight alkaline phosphatase (87%) performing best in this respect. However, measurement of high molecular weight enzymes does not offer marked advantages over more conventional enzyme tests in the detection of hepatic metastases of colorectal cancer.

The place of measurements of enzyme activities in serum as a non-invasive method of pre-operative screening for hepatic metastases in patients with colorectal and other cancers has been the subject of numerous reports (Beck *et al.*, 1979, Tartter *et al.*, 1981; Cooper *et al.*, 1975; Kemeny *et al.*, 1982). Alkaline phosphatase (EC 3.1.3.1; ALP) and γ-glutamyl transferase (EC. 2.3.2.2; GGT) are generally considered to be the most sensitive tests in current use (Huguier and Lacaine, 1981; Read *et al.*, 1977; Aronsen *et al.*, 1970). However, many patients with hepatic metastases have normal levels of these enzymes while some patients without detectable metastases have elevated levels.

In a high proportion of patients with elevated serum ALP due to cholestatic liver disease a high molecular weight form of ALP is present, and this form of ALP has been reported to be almost invariably present in sera from patients with hepatic metastases (Viot *et al.*, 1979; Crofton *et al.*, 1979). A considerable part of the high molecular weight ALP fraction (also known as the 'fast liver' or 'biliary' phosphatase) has been shown to consist of cell-membrane fragments, with which are associated other membrane-bound enzyme activities such as GGT (De Broe *et al.*, 1975). As well as occurring in serum in the form of membrane fragments ('koinozymes'), other high molecular weight fractions of GGT, and possibly also of ALP, may occur in serum as a result of such processes as aggregation of enzyme molecules with other enzymic or non-enzymic substances (Echetebu & Moss, 1982). It is possible that destructive lesions of the liver, such as those caused by malignant infiltration, may promote the disintegration of cellular membranes, thus causing the reportedly high incidence of high molecular weight enzyme fractions in serum.

We now report the results of a study of the occurrence of high molecular weight ALP and GGT in sera in patients with colorectal cancer. The value of these fractions as indicators of hepatic metastases has been compared with that of measurements of total ALP and GGT, and with specific measurement of liver type ALP (L-ALP).

### Patients and methods

Forty-two consecutive patients with colorectal cancer were studied. The 42 patients ranged in age from 43–81 years (mean age = 62 years). There were 19 men and 23 women. Thirty three patients had primary lesions in the left colon or rectum and 9 patients had primary lesions in the right colon or caecum. All patients had preoperative serum estimations of total ALP, liver isoenzyme of ALP (L-ALP) and high molecular weight ALP (H-ALP), and total GGT and high molecular weight GGT (H-GGT). Every patient subsequently underwent laparotomy within 1 week of blood sampling. At laparotomy, bimanual palpation of the liver was performed, care being taken to palpate and examine the entire liver. Needle biopsy of every suspicious lesion was performed. Liver metastases were detected by bimanual palpation of the liver in 16 patients (38%).

Correspondence: D.W. Moss.
Received 13 November 1985; and in revised form, 16 December 1985.

© The Macmillan Press Ltd., 1986

Measurements of the high molecular weight enzymes were also carried out on sera from 16 normal subjects with a similar age-range to that of the patients.

Enzyme measurements were performed at 37°C on a LKB Produkter AB 8600 reaction rate analyzer. The methods used for measurement of total ALP and total GGT were those recommended by the Committee on Enzymes of the Scandinavian Society for Clinical Chemistry and Clinical Physiology (1971, 1976 respectively). H-ALP and H-GGT activities were measured by the same methods after separation by gel filtration (Figure 1) on a Sepharose 6B column (Pharmacia, Uppsala, Sweden) of sera stored at 4°C for less than 5 days. Liver alkaline phosphatase was measured by the progressive heat-inactivation procedure of Moss & Whitby (1975). The precision of the enzyme assays was within 8% (c.v.) at normal levels and within 5% (c.v.) at elevated levels.

**Figure 1** Examples of gel-filtration profiles, showing presence of high molecular weight alkaline phosphatase (H-ALP; solid line) and high molecular weight γ-glutamyltransferase (H-GGT; broken line) in (a) normal serum, and sera from patients (b) without and (c) with metastases. High molecular weight fractions are those eluting to the left of the arrows.

The upper reference values for total ALP and L-ALP vary according to age (Whitaker *et al.*, 1977) and the upper reference values for GGT vary according to sex (Rosalki, 1975). For this reason, results are presented graphically as multiples of the upper reference value rather than as absolute values. Upper reference values for H-ALP and H-GGT were derived from data obtained for normal subjects, since these have not been established previously.

All results were analyzed statistically and the significance of differences between patients with metastases and patients without metastases was verified by the Mann–Whitney test for non-parametric data.

### Results

Total ALP was elevated in 9 of 16 patients with liver metastases and 3 of 26 patients without metastases. Although the means of the groups were significantly different ($P<0.003$), the results for the two groups overlapped considerably (Figure 2). Liver ALP was elevated in the same 9 patients with metastases and also in 5 without metastases. Again, although the difference between means was highly significant ($P<0.002$), the groups overlapped extensively (Figure 2).

**Figure 2** Total ALP, liver-specific ALP (L-ALP) and total GGT in sera of patients with colorectal cancer, with ('Mets') and without ('No mets') palpable hepatic metastases.

Total GGT showed a similar pattern. Levels were abnormal in 9 of 16 patients with metastases and in 7 of 26 patients without metastases, with a significant difference in means ($P=0.005$) but with extensive overlap (Figure 2).

Both high molecular weight ALP and high molecular weight GGT were detectable in the sera of normal subjects after gel filtration. The range of values was from 3.5 to 11.2 U l$^{-1}$ for H-ALP, and 0.7 to 4.5 U l$^{-1}$ for H-GGT. The data for both of these tests were found to fit well to log-normal distributions, and upper (97.5%) reference values calculated accordingly were $12\,U\,l^{-1}$ for H-ALP and $6\,U\,l^{-1}$ for H-GGT. These values were used to interpret the activities of these components in sera from the patients.

H-ALP was significantly greater ($P=0.002$) in the patients with metastases than in those without. However, individual values again overlapped (Figure 3). Fourteen of 16 cancer patients with metastases and 13 of 26 without metastases had elevated levels. Results for H-GGT were rather similar, with values of H-GGT being significantly greater in the group with metastases than in the group without metastases ($P<0.008$). Values in the two groups of patients overlapped (Figure 3). Eleven of 16 cancer patients with metastases had elevated levels, as had 10 of 26 without metastases.

**Figure 3** Levels of H-ALP and H-GGT in sera of patients with colorectal cancer, with ('Mets') and without ('No mets') palpable hepatic metastases. Broken lines indicate upper reference values.

No improvement in discrimination between groups of patients with and without metastases was obtained by expressing H-ALP or H-GGT as percentages of the respective total enzyme activities. Similarly, discriminant function analysis involving 2 or more enzyme tests did not improve discrimination between the groups.

### Discussion

Pre-operative detection of hepatic metastases in patients with colorectal cancer allows important planning decisions to be made regarding additional procedures which may be required, e.g. hepatic resection (Fortner *et al.*, 1984*a;* Adson & Van Heerden, 1980) or placement of catheters for intra- or post-operative perfusion chemotherapy (Fortner *et al.*, 1984*b*; Ansfield & Ramirez, 1978). The most reliable pre-operative test for hepatic metastases currently available is computer-assisted tomography with detection rates of 85–95% (Snow *et al.*, 1979; Smith *et al.*, 1982) compared with 65–85% for ultrasound scans, isotope scans and angiography (Knopf *et al.*, 1982; Bondestam *et al.*, 1980; Kim *et al.*, 1975). However, the last two procedures are complex and invasive, so that a useful role exists for a simple non-invasive biochemical screening test which would alert the clinician to the need for further diagnostic tests.

The value of serum enzyme measurements in screening for hepatic metastases has been limited by the poor sensitivity and specificity of many of the enzymes measured in the past, e.g., aspartate and alanine aminotransferases, lactate dehydrogenase (Castagna *et al.*, 1972; Schaefer & Schiff, 1965). ALP and GGT have consistently demonstrated superior sensitivity to these enzymes, but ALP may be elevated due to bone disease, and both ALP and GGT may be elevated in hepatic diseases other than metastases. The specificity and sensitivity of ALP as an indicator of liver involvement can be improved by measuring the liver-derived form of the enzyme (L-ALP). GGT is highly specific for hepatobiliary disease, but neither GGT nor L-ALP can distinguish between malignant infiltration and other liver diseases. The hypothesis that H-ALP and H-GGT may have some degree of specificity for liver metastases, because of the destructive nature of the lesions, is therefore worth investigation.

The present results do not demonstrate any marked advantage of H-GGT or H-ALP over other enzyme tests in the detection of hepatic metastases (Table I). Their efficiencies (sum of true positive and true negative results as a percentage of all patients) are rather lower than those of the other tests. This derives from the higher proportion of false positives (raised levels in patients without metastases) given by H-GGT and H-ALP; for the same reason, the predictive value of a positive

**Table I** Efficiencies and predictive values of positive and negative results of various enzyme tests in the detection of hepatic metastases of colorectal cancer

| | | *Predictive value of* | |
|---|---|---|---|
| *Test* | *Efficiency* | *Positive result* | *Negative result* |
| Total ALP | 76 | 75 | 77 |
| Total GGT | 67 | 56 | 73 |
| L-ALP | 71 | 64 | 75 |
| H-ALP | 64 | 52 | 87 |
| H-GGT | 67 | 55 | 77 |

result is lower for H-GGT and H-ALP than the other tests.

The predictive value of a negative result is similar for all the tests other than H-ALP, which performs rather better in this respect than other tests. However, these assessments depend markedly on the levels chosen to discriminate between normal and abnormal results, and the reference limits for H-ALP and H-GGT are based on fewer data than those for the other tests. A similar incidence of false positive H-ALP values (20%) in patients without liver metastases, with a high incidence (79%) of abnormal values in patients with metastases, has been reported in a study of patients with breast cancer (Karmen *et al.*, 1984).

The similarity between results for total GGT and H-GGT, and L-ALP and H-ALP, may indicate that the processes which lead to the release of these enzymes from liver cells may be similar in the absence and presence of palpable metastases; i.e. destruction of hepatocytes by invading tumour cells may not be necessary for membrane fragments to be released. An important factor in shedding of membrane fragments due to metastases may be local cholestasis (e.g., leading to elution of membrane components through detergent action). Local cholestasis may occur even before discrete metastases are palpable, and this may account for the high proportion of positive results by all tests in patients in whom no metastases could be felt. In the 20 patients without palpable liver metastases in whom follow-up was possible, 14 remained free of metastases over periods of up to 36 months (mean 22 months) and 3 had local recurrence without metastases, while 3 developed liver metastases after intervals of 4, 15 and 13 months. Of the last three patients, the first 2 had markedly elevated levels of H-GGT and H-ALP when first examined; whether high levels of these enzyme forms suggest an increased risk of hepatic involvement therefore seems to merit further investigation.

The low levels of H-ALP and H-GGT present in normal sera were not correlated ($r = 0.06$) suggesting that, in these samples, different aggregates of the two enzymes are present. However, in patients (with or without palpable liver metastases) the levels were strongly correlated ($r = 0.89$), as would be expected as a result of the release of cell membrane fragments carrying both ALP and GGT. It is not possible at present to determine whether the specific activities of the complexed enzymes are reduced compared with those of lower molecular weight forms, and therefore whether activity measurements underestimate the relative amounts of complexed enzymes present in serum.

The work presented in this paper was partly supported by the Cancer Research Campaign.

### References

ADSON, M.A. & VAN HEERDEN, J.A. (1980). Major hepatic resections for metastatic colorectal cancer. *Ann. Surg.*, **191,** 576.

ANSFIELD, F.J. & RAMIREZ, G. (1978). The clinical results of 5 fluorouracil intrahepatic arterial infusion in 528 patients with metastatic cancer to the liver. *Prog. Clin. Cancer*, **7,** 217.

ARONSEN, K.F., NOSSLIN, B & PIHL, B. (1970). The value of γ-glutamyl transpeptidase as a screen test for liver tumour. *Acta Chir. Scand.*, **136,** 17.

BECK, P.R., BELFIELD, A., SPOONER, R.J., BLUMGART L.H. & WOOD, C.B. (1979). Serum enzymes in colorectal cancer. *Cancer* **43,** 1772.

BONDESTAM, S., LAHDE, S., ANNALA, R., AANTAA, K. & PERTTALA, Y. (1980). Scintigraphy and sonography in the investigation of liver metastases. *Diagn. Imaging*, **49,** 339.

CASTAGNA, J., BENFIELD, J.R., YAMADA, H. & JOHNSON, D.E. (1972). The reliability of liver scans and function tests in detecting metastases. *Surg. Gynecol. Obstet.*, **134,** 463.

Committee on Enzymes of the Scandinavian Society for Clinical Chemistry and Clinical Physiology (1971). Recommended methods for the determination of four enzymes in blood. *Scand. J. Clin. Lab. Invest.*, suppl., **118,** 291.

Committee on Enzymes of the Scandinavian Society for Clinical Chemistry and Clinical Physiology (1976). Recommended method for the determination of γ-glutamyl transferase in blood. *Scand. J. Clin. Lab. Invest.*, **36,** 119.

COOPER, E.H., TURNER, R., STEELE, L., NEVILLE, A.M. & MACKAY, A.M. (1975). The contribution of serum enzymes and carcinoembryonic antigen to the early diagnosis of metastatic colorectal cancer. *Br. J. Cancer*, **31,** 111.

CROFTON, P.M., ELTON, R.A. & SMITH, A.F. (1979). High molecular weight alkaline phosphatase: a clinical study. *Clin. Chim. Acta* **98,** 263.

DE BROE, M.E., BORGERS, M. & WIEME, R.J. (1975). The separation and characterization of liver plasma membrane fragments circulating in the blood of patients with cholestasis. *Clin. Chim. Acta*, **59,** 369.

ECHETEBU, Z.O. & MOSS, D.W. (1982). Multiple forms of human γ-glutamyltransferase. Preparation and characterization of different molecular weight fractions. *Enzyme*, **27,** 1.

FORTNER, J.G., SILVA, J.S., GOLBEY, R.B., COX, E.B. & MACLEAN, B.J. (1984*a*). Multivariate analysis of a personal series of 247 consecutive patients with liver metastases from colorectal cancer: treatment by hepatic resection. *Ann. Surg.*, **199,** 306.

FORTNER, J.G., SILVA, J.S., COX, E.B., GOLBEY, R.B., GALLOWITZ, H. & MACLEAN, B. (1984*b*). Multivariate analysis of a personal series of 247 patients with liver metastases from colorectal cancer: treatment by intrahepatic chemotherapy. *Ann. Surg.*, **199**, 317.

HUGUIER, M. & LACAINE, F. (1981). Hepatic metastases in gastrointestinal cancer – diagnostic value of biochemical investigations. *Arch. Surg.*, **116**, 399.

KARMEN, C., MAYNE, P.D., FOO, A.Y., PARBHOO, S. & ROSALKI, S.B. (1984). Measurement of biliary alkaline phosphatase by minicolumn chromatography and by electrophoresis and its application to the detection of liver metastases in patients with breast cancer. *J. Clin. Pathol.*, **37**, 212.

KEMENY, M.M., SUGARBAKER, P.H., SMITH, T.J. & 4 others (1982). A prospective analysis of laboratory tests and imaging studies to detect hepatic lesions. *Ann. Surg.*, **195**, 163.

KIM, D.K., McSWEENEY, J., YEM, S.D.J. & FORTNER, J.G. (1975). Tumours of the liver as demonstrated by angiography, scan and laparotomy. *Surg. Gynecol. Obstet.*, **141**, 409.

KNOPF, D.R., TORRES, W.E., FAJMAN, W.J. & SONES, P.J. (1982). Liver lesions: comparative accuracy of scintigaphy and computed tomography. *Am. J. Roentgen.*, **138**, 623.

MOSS, D.W. & WHITBY, L.G. (1975). A simplified heat-inactivation method for investigating alkaline phosphatase isoenzymes in serum. *Clin. Chim. Acta*, **61**, 63.

READ, D.R., HAMBRICK, E., ANCARIAN, H. & LEVINE, H. (1977). The preoperative detection of hepatic metastases in cases of colorectal carcinoma. *Dis. Col. Rect.*, **20**, 101.

ROSALKI, S.B. (1975). Gamma-glutamyl transpeptidase. *Adv. Clin. Chem.*, **17**, 53.

SCHAEFER, J. & SCHIFF, L. (1965). Liver function tests in metastatic tumour of the liver: study of 100 cases. *Gastroenterology*, **49**, 360.

SMITH, T.J., KEMENY, M.M., SUGARBAKER, P.H. & 4 others (1982). A prospective study of hepatic imaging in the detection of metastatic disease. *Ann. Surg.*, **195**, 486.

SNOW, J.H., GOLDSTEIN, H.M. & WALLACE, S. (1979). Comparison of scintigraphy, sonography, and computed tomography in the evaluation of hepatic neoplasms. *Am. J. Roentgen.*, **132**, 915.

TARTTER, P.I., SLATER, G., GELERNT, I. & AUFSES, A.H. (1981). Screening for liver metastases from colorectal cancer with carcinoembryonic antigen and alkaline phosphatase. *Ann. Surg.*, **193**, 357.

VIOT, M., JOULIN, C., CAMBON, P., KREBS, B.P., SCHNEIDER, M. & LALANNE, C.M. (1979). The value of serum alkaline phosphatase $\alpha_1$ isoenzyme in the diagnosis of liver metastases. *Biomedicine*, **31**, 74.

WHITAKER, K.B., WHITBY, L.G. & MOSS, D.W. (1977). Activities of bone and liver alkaline phosphatases in serum in health and disease. *Clin. Chim. Acta*, **80**, 209.